# Effect of natural ageing and heat treatments on GII.4 norovirus binding to Histo-Blood Group Antigens

**DOI:** 10.1038/s41598-019-51750-4

**Published:** 2019-10-25

**Authors:** Maëlle Robin, Manon Chassaing, Julie Loutreul, Alexis de Rougemont, Gaël Belliot, Didier Majou, Christophe Gantzer, Nicolas Boudaud

**Affiliations:** 1 Actalia, Food Safety Department, F-50000 Saint-Lô, France; 2 LCPME, UMR 7564 CNRS, University of Lorraine, F-54601 Villers-lès-Nancy, France; 3 National Reference Center for Gastroenteritis Viruses, Laboratory of Virology, University Hospital of Dijon, Dijon, F-21000 France; 4 UMR PAM A 02.102 Food and Microbiological Processes, University of Bourgogne Franche-Comté/AgroSup Dijon, Dijon, F-21000 France; 5 ACTIA, F-75231 Paris Cedex 05, Cedex, France

**Keywords:** Microbiology techniques, Virology

## Abstract

Human noroviruses (HuNoVs) are the leading cause of viral foodborne outbreaks worldwide. To date, no available methods can be routinely used to detect infectious HuNoVs in foodstuffs. HuNoVs recognize Histo-Blood Group Antigens (HBGAs) through the binding pocket (BP) of capsid protein VP1, which promotes infection in the host cell. In this context, the suitability of human HBGA-binding assays to evaluate the BP integrity of HuNoVs was studied on GII.4 virus-like particles (VLPs) and GII.4 HuNoVs during natural ageing at 20 °C and heat treatments. Our results demonstrate that this approach may reduce the over-estimation of potential infectious HuNoVs resulting from solely using the genome detection, even though some limitations have been identified. The specificity of HBGA-binding to the BP is clearly dependent on the HGBA type (as previously evidenced) and the ionic strength of the media without disturbing such interactions. This study also provides new arguments regarding the ability of VLPs to mimic HuNoV behavior during inactivation treatments. The BP stability of VLPs was at least 4.3 fold lower than that of HuNoVs at 20 °C, whereas capsids of both particles were disrupted at 72 °C. Thus, VLPs are relevant surrogates of HuNoVs for inactivation treatments inducing significant changes in the capsid structure.

## Introduction

Human noroviruses (HuNoVs), responsible for acute gastroenteritis, are the leading cause of viral foodborne outbreaks in Europe and USA each year^1,2^. HuNoVs, which belong to the family *Caliciviridae*, are mainly genogroup I (GI) and II (GII) NoVs which are subdivided into several genotypes^3^. Genogroup II, genotype 4 (GII.4) NoV is the most prevalent, and has been causing for about 70% of outbreaks since 2002^4^. Infected individuals release high concentrations of HuNoVs in their feces, ranging from 10^6^ to 10^9^ genome copies (gc)/gram of stools over a 4- to 8-week period^5^. The infectious dose for HuNoVs has not been clearly defined yet. For Norwalk virus (NV), the 50% human infectious dose (HID_50_) was estimated between 18 and 2,800 genomic equivalents for secretor individuals and by considering the viral aggregation^6,7^. Thus, high viral shedding in the environment combined with a relatively low HID_50_ contributes to promote HuNoV transmission to the human population. HuNoVs are transmitted through the fecal-oral route, either directly by close contact with infected people or indirectly through consumption of contaminated water and food^8^.

HuNoVs are non-enveloped viruses (about 40 nm in diameter) with a capsid of icosahedral symmetry, and a single-stranded positive-sense RNA (~7,500 nucleotides) containing three open reading frames (ORFs). ORF1 encodes non-structural proteins while ORF2 and ORF3 encode for the major (VP1) and the minor (VP2) capsid proteins, respectively. The capsid is composed of 180 copies of VP1 assembled into 90 dimers and few copies of VP2. VP1 is organized into two domains, the shell domain (S) and the protruding domain (P) which is subdivided into P1 and P2 subdomains^9^.

The occurrence of HuNoV foodborne outbreaks underlines that these viruses can remain infectious for a substantial time in the environment^10–12^. The food vehicles most commonly involved in HuNoV foodborne outbreaks are raw and minimally processed foods (e.g. bivalve molluscan shellfish, fresh vegetables, drinking water). Even though no regulation exists in Europe for the management of HuNoVs, their detection in foodstuffs put on the market is on the rise for the last few years. This trend is related to the publication of the ISO 15216-1 standard in 2013, allowing the genome detection of HuNoVs in vulnerable foodstuffs^13^. Based on representative sampling, the prevalence of HuNoV genomes ranges from 9.0% to 71.6% in shellfish and from 5.0% to 28.2% in fresh produce^14^. Although its interest is unquestionable for viral outbreak investigations, the routine use of this ISO standard^13^ for surveillance in foodstuffs has limitations due to its inability to discriminate infectious from non-infectious particles when HuNoV genomes are detected^15^. This drawback is related to the fact that HuNoVs cannot be routinely cultivated in laboratories, despite recent advances for the *in vitro* replication of few strains of HuNoVs using human intestinal enteroids^16^. From our point of view, it is important to underline that even if the cellular models of HuNoVs were mastered by control laboratories over the coming years, this approach would not be suitable for routine food testing owing to its complexity, high cost, and time-consumption.

In this context, it becomes necessary to develop a suitable molecular method for discriminating infectious from non-infectious HuNoVs in food and water in order to (*i*) enable better management of the viral hazard and (*ii*) improve the effectiveness of risk management strategies in industries. To solve this critical issue, numerous strategies have been implemented over the past two decades. Firstly, a number of cultivable enteric viruses were used as HuNoV surrogates to assess the virucidal effect of disinfection treatments applied in food and water industries. Among HuNoV surrogates, feline calicivirus, murine NoV (MNV), Tulane virus (TV), and F-specific RNA bacteriophages (FRNAPH) are commonly used^8,17,18^. VLPs of HuNoVs are also used as well^8,19,20^. They are morphologically and immunologically similar to the native virions^21,22^.

Secondly, several methods have been proposed to discriminate infectious from non-infectious HuNoVs such as (*i*) the detection of the whole genome^23^, (*ii*) the use of enzymatic pre-treatments or intercalating dyes before genome detection^24,25^, and (*iii*) the detection of oxidative damages on the capsid^26^. The use of receptor binding to assess the capsid integrity is another promising approach^27–29^. Several studies have demonstrated that Histo-Blood Group Antigens (HBGAs) display a role of receptors or co-factors for HuNoVs which promote the infection in host cells^30–33^. HBGAs are complex carbohydrates linked to glycolipids or glycoproteins found on the red blood cells and mucosal epithelial cells. They can also be present as free antigens in biological fluids such as saliva^34^. The biosynthesis of HBGAs is governed by the ABO and the fucosyl-transferase *FUT2* and *FUT3* gene families^34,35^. The expression of active or non-active *FUT2* defines the secretor or non-secretor status, respectively^32,35^.

The HuNoV-binding site to HBGAs is located in a region of the P2 subdomain of VP1 called “binding pocket” (BP)^36^. Interactions between HuNoVs and HBGAs have been described to be very specific with multiple binding patterns and variable relative affinities^34,37,38^. Thus, the human susceptibility to HuNoV infection is dependent on both polymorphic HBGA expression and HuNoV genotype^30,33,39,40^. According to these findings, some authors have made the assumption that only HuNoV particles able to bind the HBGAs would infect host cells, and thus potentially allowing discrimination infectious from non-infectious particles^41^.

The aim of this study was to investigate the ability of GII.4 VLPs to mimic the HuNoV behavior and the suitability of the HBGA-binding assays to avoid the over-estimation of potential infectious HuNoV number given by genome detection during natural ageing at 20 °C over time and heat treatments (50 °C, 60 °C, and 72 °C) by using the most prevalent HuNoV genotype worldwide (i.e. GII.4). First, the capsid integrity of GII.4 VLPs was studied during these inactivation treatments using receptor-binding enzyme linked immunosorbent assays (ELISA) and transmission electronic microscopy (TEM). The influence of the ionic strength of the medium on VLP binding to HBGAs was explored to assess the VLP stability and the nature of interactions between VLPs and HBGAs. Then, the HBGA-binding capacity of intact GII.4 HuNoVs extracted from human stools was evaluated using HBGA-binding followed by RNA amplification by quantitative RT-PCR (RT-qPCR) under the same conditions as those used with GII.4 VLPs. Finally, we compared the representativeness of using VLPs as HuNoV surrogates for a same genotype during inactivation treatments. We also discussed the benefits of using HBGA-binding assays to assess the capsid integrity and the suitability of this strategy to indicate the infectivity of HuNoVs after inactivation treatments.

## Results

### Binding profile of GII.4 VLPs to saliva samples

HBGA types were determined for the 27 human saliva samples by ELISA and were distributed as follows: non-secretor phenotypes (5/27; 18.5%), A Lewis-negative (ALe^−^) (1/27; 3.70%), A Lewis-positive (ALe^+^) (8/27; 29.6%), BLe^−^ (1/27; 3.70%), BLe^+^ (4/27; 14.8%), and OLe^+^ (8/27; 29.6%). OLe^−^ type was not found in the tested saliva samples. This distribution is consistent with the prevalence of 20% of non-secretor individuals in the Caucasian population^37^.

The binding profile of GII.4 VLPs to all saliva samples was performed using HBGA-binding ELISA (see Supplementary Fig. S1). Based on the optical density (OD_450_) values obtained under the same conditions, VLPs recognized all HBGAs from secretor individuals (22/27; 81.5%). Conversely, no VLP binding was observed for the five non-secretor saliva samples (positive/negative (P/N) ratio < 2). These results are consistent with published data^22,37^. Nevertheless, variable relative affinities were highlighted regarding interactions between VLPs and saliva samples from secretor individuals. This could be explained by the amount of HBGAs in each saliva^37^. The extent of VLP binding was particularly high for one secretor saliva corresponding to OLe^+^ type (OD_450_ = 0.625 at 1 µg/mL of GII.4 VLPs). Consequently, this sole saliva sample was used for all experiments performed in this study to evaluate the effect of natural ageing at 20 °C and heat treatments (50 °C, 60 °C, and 72 °C) on the NoV binding capacity.

### Sensitivity and linearity of the HBGA-binding ELISA

The sensitivity and the linearity of the HBGA-binding ELISA method was investigated using OLe^+^ saliva and various concentrations of GII.4 VLPs ranging from 0.05 and 10.0 µg/mL in 10 mM and 150 mM PBS solutions (see Supplementary Fig. S2). The limit of detection (LOD) was estimated at 0.10 µg/mL of GII.4 VLPs because the P/N ratio was lower than 2 below this threshold value under the two ionic strength conditions. The relationship between OD_450_ and the VLP concentration was not linear in the whole range especially above a threshold value corresponding at 1.00 µg/mL, due to the concentration of anti-GII.4 VLP monoclonal antibody (MAb) which becomes limiting. In tested conditions for natural ageing and heat treatments (10.0 µg/mL of GII.4 VLPs), the first log_10_ reduction of HBGA-binding does not have a substantial impact on the OD_450_ values. Conversely, the first 2 log_10_ reductions lead to a decrease of OD_450_ values until the LOD. So, we considered that 2 log_10_ reductions (T_99_) were obtained when OD_450_ values were equal to the LOD.

### Effect of natural ageing at 20 °C and heat treatments on the GII.4 VLP binding to human HBGAs and on the capsid integrity

**Figure 1:**
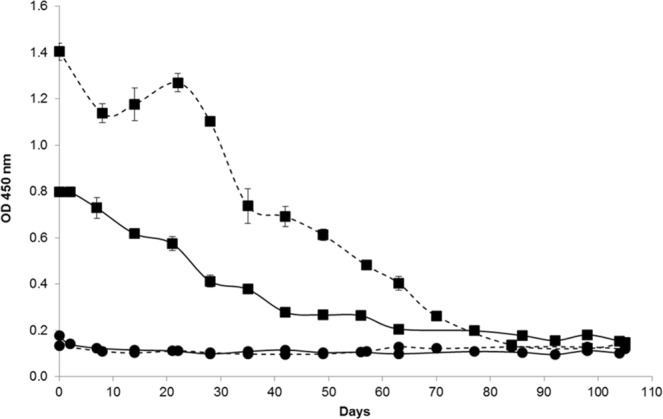
GII.4 VLP binding to OLe^+^ saliva after exposure to natural ageing at 20 °C under low (10 mM PBS) and high (150 mM PBS) ionic strength conditions. The OD_450_ values of purified GII.4 VLPs were obtained by HBGA-binding ELISA in 10 mM solution (solid line) and 150 mM PBS solution (dashed line). Squares (▪) and circles (•) represent the VLP binding to untreated and sodium periodate-treated OLe^+^ human saliva, respectively. Each data point represents the mean OD_450_ values of four replicates and error bars indicate standard deviations. Paired and unpaired Student’s *t*-test was used to compare groups.

The effect of natural ageing at 20 °C over 105 days under two ionic strength conditions (10 mM and 150 mM PBS solutions) on GII.4 VLP binding to OLe^+^ saliva is shown in Fig. 1. For each assay, a sodium periodate treatment on OLe^+^ saliva was added as a negative control to check the specificity of VLP-HBGA interactions. For all trials, no VLP binding was observed after sodium periodate treatment (OD_450_ < 0.200) due to the oxidative cleavage of saccharide rings^42^. According to the OD_450_ values, a progressive reduction in VLP binding to OLe^+^ saliva was observed over time until reaching a complete loss of binding capacity after 63 and 70 days under low (10 mM PBS) and high (150 mM PBS) ionic strength conditions, respectively (P/N ratio < 2). From these days, the LOD was reached, consequently, 2 log_10_ reductions of the OD_450_ values were obtained. Under the high ionic strength condition, no significant difference was observed after 70 days between untreated and sodium periodate-treated samples (paired sample *t*-test, *p* > 0.26). Under the low ionic strength condition, after 63 days, differences between untreated and sodium periodate-treated saliva samples appeared at the limit of significance (paired sample *t*-test, *p* < 0.0021). Based on the GII.4 VLP binding profiles, natural ageing at 20 °C affects gradually the BP integrity. However, changes in the magnitude of the binding profile were highlighted as a function of the ionic strength. For the first 63 days, the relative affinity of the VLP binding was significantly higher at high ionic strength compared to low ionic strength (unpaired sample *t*-test, *p* < 10^−4^). Between 70 and 98 days, the VLP binding was low and statistically different between the two ionic strength conditions (unpaired sample *t*-test, *p* < 10^−3^). After 105 days, no statistically significant difference was observed between the two ionic strength conditions (unpaired sample *t*-test, *p* > 0.25).

**Figure 2:**
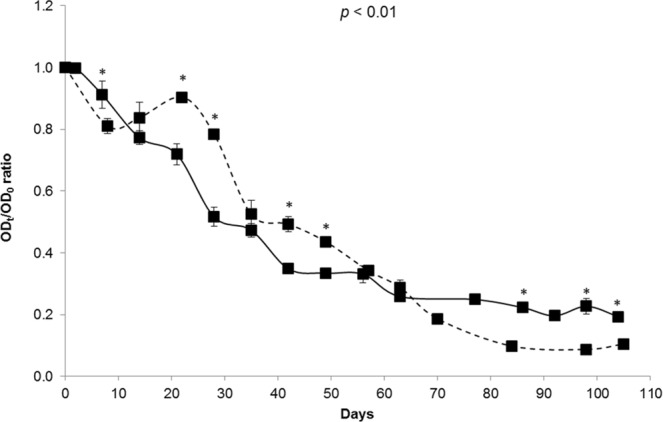
Decay of GII.4 VLP binding to OLe^+^ saliva after exposure to natural ageing at 20 °C under low and high ionic strength conditions. The solid and dashed lines represent purified GII.4 VLP binding to untreated saliva under low (10 mM PBS) and high (150 mM PBS) ionic strength conditions, respectively. The OD_t_/OD_0_ ratio was determined by dividing the mean OD_450_ values obtained by HBGA-binding ELISA at each time point (OD_t_) by the mean OD_450_ values obtained at day 0 (OD_0_). Each data point represents the mean OD_450_ values of four replicates and error bars indicate standard deviations. Unpaired Student’s *t*-test was used to compare the two groups.

To better evaluate the influence of the ionic strength on GII.4 VLP binding to OLe^+^ saliva, the OD_t_/OD_0_ ratio was determined in 10 mM and 150 mM PBS solutions (Fig. 2). Similar trends were observed regarding the reduction in VLP binding during natural ageing at 20 °C. No significant difference was observed between the two ionic strength conditions after 14, 35, 56, and 63 days (unpaired sample *t*-test, *p* > 0.07). For the others points, significant differences appeared (unpaired sample *t*-test, *p* < 0.01). The ionic strength of the medium seems to influence the magnitude of the binding profile but without disturbing the specificity of VLP-HBGA interactions. Consequently, the binding profiles of GII.4 VLPs to OLe^+^ saliva after inactivation by heating were only performed under the high ionic strength condition.

**Figure 3:**
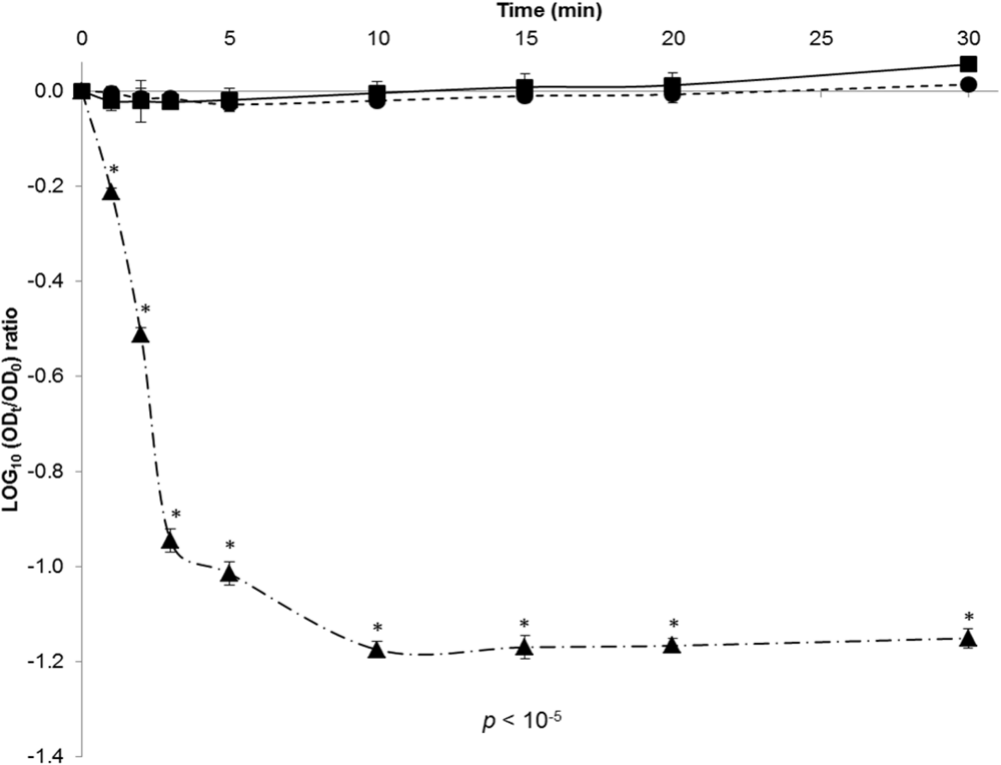
Decay of GII.4 VLP binding to OLe^+^ saliva after exposure to heat treatments under high ionic strength condition (150 mM PBS). Squares (▪, solid line), circles (•, dotted line), and triangles (▲, dot-and-dash line) represent purified GII.4 VLP binding to untreated OLe^+^ human saliva after heating at 50 °C, 60 °C, and 72 °C for 30 min, respectively. The log (OD_t_/OD_0_) values were determined by dividing the mean OD_450_ values obtained by HBGA-binding ELISA at each time point (OD_t_) by the mean OD_450_ values obtained at time 0 (OD_0_). Each data point corresponds to the mean OD_450_ values of triplicates and error bars indicate standard deviations. Paired and unpaired Student’s *t*-test was used to compare groups.

The log_10_ reductions in GII.4 VLP binding to OLe^+^ saliva after exposure to heat treatments (50 °C, 60 °C, and 72 °C) for 30 min were represented by the log (OD_t_/OD_0_) at 450 nm (Fig. 3). A sodium periodate treatment was also introduced as a negative control for all assays (data not shown). No significant reduction in the VLP binding was observed after heating at 50 °C and 60 °C for 30 min (paired sample *t*-test, *p* > 0.05). The behavior of VLP binding was not statistically different for these two heat treatments (unpaired sample *t*-test, *p* > 0.2). Because of the non-linear relationship between OD_450_ values and GII.4 VLP concentration, it is only possible to consider that after 30 min at 50 °C and 60 °C, the reduction of the VLP binding to OLe^+^ saliva is lower than 1 log_10_. Conversely, a significant reduction in the VLP binding was observed at 72 °C for the first 3 min until reaching a complete loss of the OD_450nm_ detection after 10 min (paired sample *t*-test, *p* < 10^−5^). After 2 min at 72 °C, 2 log_10_ reductions were achieved because OD_450_ values were equal to the LOD. According to the log_10_ reduction profiles, temperatures below 60 °C for 30 min affect slightly the BP integrity whereas 72 °C can be considered as a critical temperature which disturbs the BP from the early stage of treatment.

**Figure 4:**
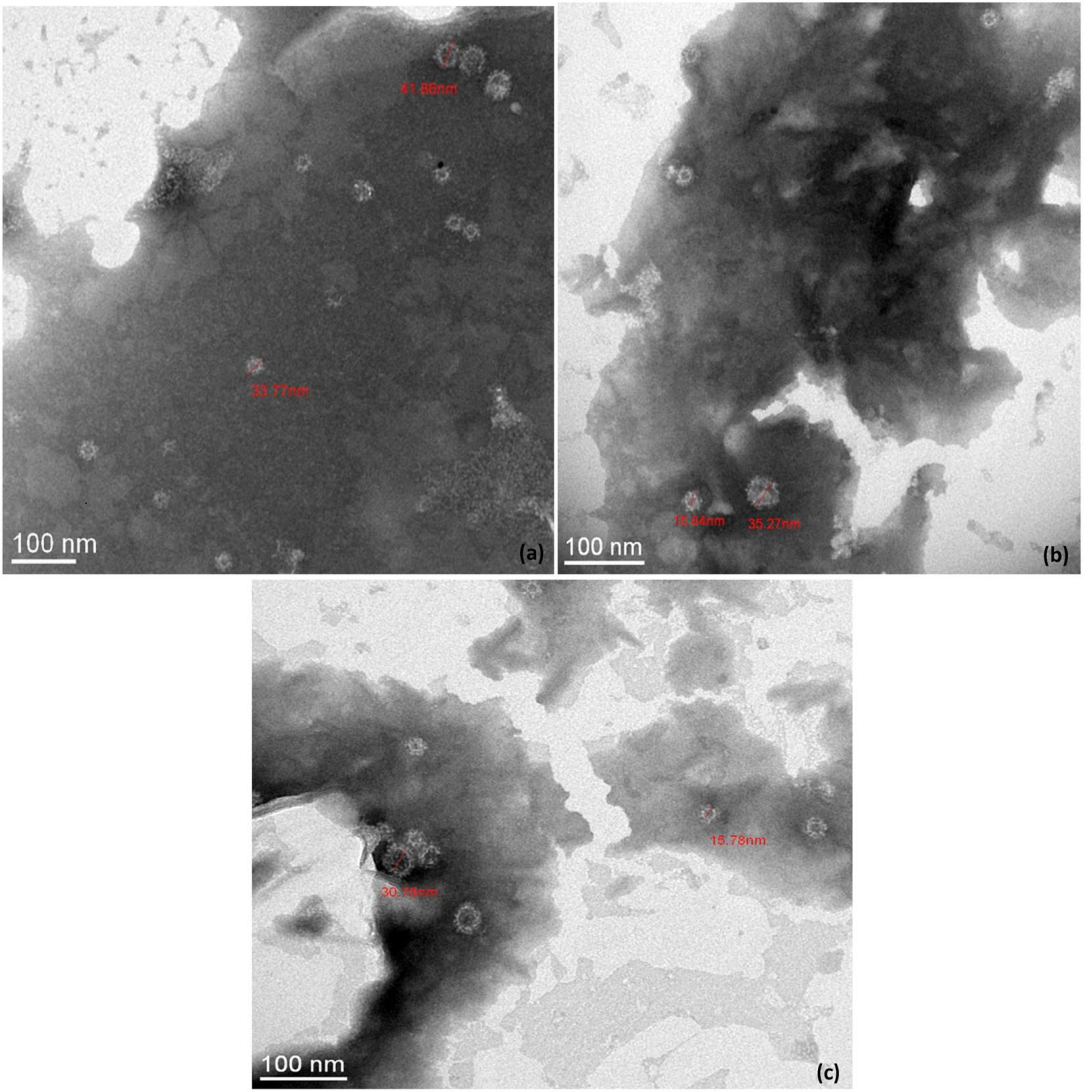
TEM observations of native and heat-treated GII.4 VLP capsids after negative staining. (**a**) Native GII.4 VLPs, **(b)** GII.4 VLPs after exposure at 50 °C for 10 min, and **(c)** GII.4 VLPs after exposure at 60 °C for 10 min. No GII.4 VLP was observed by TEM after heating at 72 °C for 10 min. The scale bar is at the bottom left of each picture.

TEM observations were performed to evaluate the influence of heat treatments on the overall structure of GII.4 VLPs (Fig. 4). Similar structures were observed for native and treated VLPs after heating at 50 °C and 60 °C for 10 min, supporting structural integrity of the capsid. Conversely, no VLP was observed after heating at 72 °C for 10 min, suggesting disruption and/or denaturation of the capsid. Two different sizes (15 and 40 nm) of GII.4 VLP populations were distinguished at 50 °C and 60 °C. Particles of 15 and 40 nm are respectively formed of 60 and 180 copies of VP1 which are described to be morphologically and antigenically similar^43^. According to these data, TEM results are consistent with the log_10_ reduction profiles of the VLP binding after heat treatments.

### Effect of natural ageing at 20 °C and heat treatments on the GII.4 HuNoV binding to human HBGAs and on the viral genome

**Figure 5:**
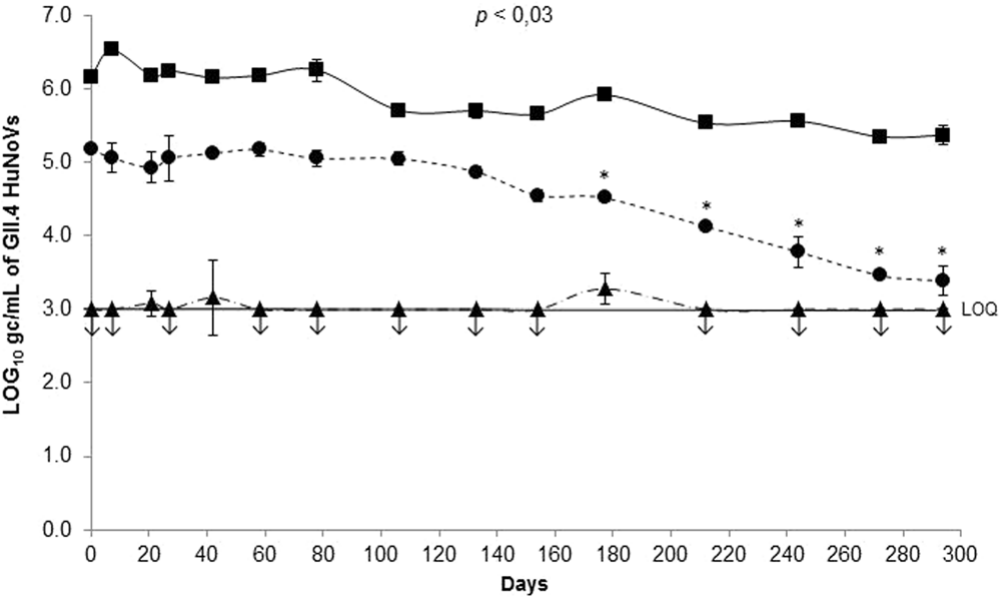
Persistence of GII.4 HuNoV genomes and GII.4 HuNoV binding to OLe^+^ saliva after exposure to natural ageing at 20 °C under low ionic strength condition (10 mM PBS). Quantification of purified GII.4 HuNoV gc was obtained by HBGA-binding assays followed by RNA amplification by RT-qPCR. Squares (▪, solid line) represent GII.4 HuNoV gc/mL treated with RNase. Circles (•, dotted line) and triangles (▲, dot-and-dash line) represent GII.4 HuNoV gc/mL quantified after capsid binding to untreated and sodium periodate-treated OLe^+^ human saliva, respectively. Each data point is an average of two replicates and error bars indicate standard deviations. Arrows (↓) represent data below the LOQ but above the LOD. The LOD and LOQ were respectively 200 and 1,000 gc/mL of purified GII.4 HuNoVs. Paired Student’s *t*-test was used to compare groups.

The decay curves of the GII.4 HuNoV genomes treated with ribonuclease A (RNase A) and the binding capacity of viral particles to OLe^+^ saliva with or without sodium periodate treatment after exposure to natural ageing at 20 °C for 294 days in 10 mM PBS solution are shown in Fig. 5. No significant reduction in the RNase-treated genomes of HuNoVs was observed during the whole treatment (<1.2 log_10_) (paired sample *t*-test, *p* > 0.05) suggesting the persistence of potentially intact capsids because the genomes were not accessible to RNase A. Based on linear regression, the T_90_ was estimated at 294 days. During the whole treatment, a fraction of purified HuNoVs (mainly ranging between the LOD and the limit of quantification (LOQ) of the method) were able to bind to OLe^+^ saliva treated with sodium periodate, suggesting non-specific interactions between capsids and denatured HBGAs. Concerning GII.4 HuNoV binding to untreated OLe^+^ saliva, no significant variation was observed for the first 154 days with genome detection rates ranging from 4.50 to 5.20 log_10_ (paired sample *t*-test, *p* > 0.05). A 1.2 log_10_ reduction in the binding capacity was shown between day 154 and day 294, achieving almost the LOQ determined at 1,000 gc/mL (paired sample *t*-test, *p* < 0.03). Based on linear regression over the whole time period, the T_90_ was estimated at 166 days.

**Figure 6:**
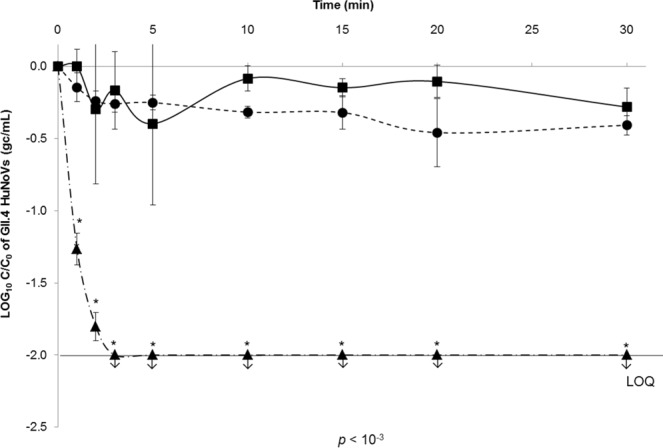
Decay of GII.4 HuNoV binding to OLe^+^ saliva after exposure to heat treatments under high ionic strength condition (150 mM PBS). Squares (▪, solid line), circles (•, dotted line), and triangles (▲, dot-and-dash line) represent purified GII.4 HuNoV binding to untreated OLe^+^ human saliva after heating at 50 °C, 60 °C, and 72 °C for 30 min, respectively. The log (C/C_0_) values were calculated by dividing the mean GII.4 HuNoV gc/mL values obtained by HBGA-binding assays followed by RNA amplification by RT-qPCR at each time point (C_t_) by the mean GII.4 HuNoV gc/mL values obtained at time 0 (C_0_). Each data point is an average of three replicates and error bars indicate standard deviations. Arrows (↓) represent data below the LOQ but above the LOD. The LOD and LOQ were 200 and 1,000 gc/mL of purified GII.4 HuNoVs, respectively. Paired Student’s *t*-test was used to compare groups.

As performed for GII.4 VLPs under the same conditions, the log_10_ reductions in GII.4 HuNoV binding to OLe^+^ saliva after exposure to heat treatments (50 °C, 60 °C, and 72 °C) for 30 min were determined using the log (C/C_0_) (Fig. 6). A sodium periodate treatment was also added as a negative control for all assays (data not shown). No significant reduction in the binding capacity was observed after heating at 50 °C and 60 °C for 30 min (paired sample *t*-test, *p* > 0.05). Conversely, a statistically significant reduction was shown at 72 °C after 2 min until a complete loss of the genome detection after 3 min (paired sample *t*-test, *p* < 10^−3^). As described for VLPs, temperatures below 60 °C for 30 min affect slightly the BP, whereas 72 °C seems to impair the BP integrity. Interestingly, the behavior of viral capsids of GII.4 VLPs and HuNoVs seems to be similar after heat treatments in the tested conditions (Figs 3 and 6). Depending on the contact time, a critical temperature above which the viral capsid is disrupted, could be given between 60 °C and 72 °C.

### Comparison of the loss of GII.4 VLP and HuNoV binding to OLe^+^ saliva after inactivation treatments

Because of the absence of linear relationship between OD_450_ values and the content of GII.4 VLPs, the time to reach the first 2 log_10_ reductions of HBGA-binding (T_99_) was used to compare the loss of GII.4 VLP and HuNoV binding to OLe^+^ saliva after natural ageing at 20 °C and heating at 72 °C (Table 1). For VLPs, T_99_ values were estimated between T_0_ (10 µg/mL) and the first time point when the LOD was reached (0.1 µg/mL). For HuNoVs, T_99_ values were derived from the slope of the linear regression analysis. After natural ageing at 20 °C, the T_99_ value of HuNoVs (333 days) is at least 4.3 fold higher than that of VLPs (ranging between 63 and 77 days) under the low ionic strength condition, demonstrating a different behavior of GII.4 VLPs and HuNoVs. Considering the similarity of the T_99_ values of VLPs at 10 mM and 150 mM PBS solutions, this result reinforces the fact that the ionic strength of the medium does not disturb the specificity of VLP-HBGA interactions. After heating at 72 °C, the T_99_ values were similar for GII.4 HuNoVs and VLPs and estimated at 0.002 days and ranging between 0.001 and 0.002 days, respectively.

**Table 1:** Time to achieve 2 log_10_ reductions of HBGA-binding (T_99_) for GII.4 VLPs and HuNoVs after inactivation treatments.

Inactivation treatments	T_99_ values (days)	
	GII.4 VLPs	GII.4 HuNoVs
72 °C (150 mM PBS)	[0.001–0.002]	0.002
Natural ageing (10 mM PBS)	[63–77]	333
Natural ageing (150 mM PBS)	[70–84]	ND

For GII.4 VLPs, T_99_ values were estimated between T_0_ (10 µg/mL) and the first time point when the LOD was reached (0.1 µg/mL) for the inactivation treatments that led to at least 2 log_10_ reductions. For GII.4 HuNoVs, T_99_ values were derived from the slope of the linear regression analysis during the whole duration of inactivation treatments, excepted after heating at 72 °C for which the linear relationship was comprised between 0 and 2 min.

ND: non determined.

## Discussion

For the past two decades, many studies have tried to develop the most reliable strategy to distinguish infectious from non-infectious HuNoVs in foodstuffs and in the environment with the aim to improve the management of HuNoV hazard but also to better assess the effectiveness of inactivation treatments. These efforts have increased even more since the publication of the ISO 15216-1 standard^13^ in 2013 because of its inability to provide information about HuNoV infectivity when their genome is detected in vulnerable foodstuffs in prospective approaches such as surveys. Among all those proposed, the strategy based on the receptor/co-factor (HBGAs) binding to the BP to assess the capsid integrity of HuNoVs appeared promising^28,41^. The first objective of this study was to evaluate the ability of VLPs to mimic the behavior of HuNoVs for a same genotype (i.e. GII.4) during inactivation treatments. The second objective was to highlight the benefits/limitations of HuNoV binding to human HBGAs to potentially discriminate infectious from non-infectious particles or to prove the capsid integrity.

Our study provides new arguments to discuss whether VLPs might be suitable models to represent the behavior of HuNoVs. GII.4 VLPs are only self-assemblies of VP1 that also involve electrostatic or hydrophobic interactions in the environment^44^. Such interactions are strongly affected by the ionic strength of the medium. The results indicate that VLPs are only weakly affected by the ionic strength range of 10 to 150 mM at pH 7.4 over a long period of time at 20 °C, as observed for other complete viruses^45,46^. In the same way, the capsids of GII.4 VLPs and HuNoVs are both disrupted after heating at 72 °C, as it was observed for GII.4 VLPs at 68 °C by Moore *et al*.^41^. Ausar *et al*.^19^ have also shown that the native quaternary structure of NV is maintained until 60 °C but irreversible capsid disruptions occur above 65 °C. Structural changes in the capsid proteins and/or loss of binding affinity to host cells could occur above a critical temperature, as demonstrated for MS2 phage at 72 °C^47^.

Depending on the contact time, a critical temperature range of 60 °C to 72 °C leading to the disruption of the capsid may be proposed for VLPs as well as for HuNoVs. Surprisingly, the exact same critical temperature range has also been suggested for MNV^48^. Further research should be conducted to determine the critical contact time in the 60–72 °C temperature range.

On the other hand, our study also underlines major behavioral differences between GII.4 VLPs and HuNoVs. One difference is the size of the particles because part of VLPs have a smaller size (15 nm) than that expected for HuNoVs of about 40 nm^43^. But the main critical difference may be the stability of VLPs at 20 °C, which is at least 4.3 fold lower than that of HuNoVs. As highlighted by the T_99_ values, the reduction in VLP binding to human HBGAs was substantially more rapid than for HuNoVs under the same conditions. To our knowledge, no study has described the effect of natural ageing at 20 °C on the BP integrity of HuNoVs. Some differences may also exist at higher temperatures (above 50 °C) but the duration of treatment was not sufficient to draw any conclusions. Our results are consistent with the literature data regarding the effect of heat on GII.4 VLPs^27,28^. Hence, lesser stability of VLPs can be noted, at least in the BP region. It may be related to the absence of the viral genome or the VP2 protein which may play a role on HuNoV stability^44,49^. Dika *et al*.^50^ have shown that MS2 VLPs, which lack the genome and the maturation protein, have a capsid that expresses a different charge from that of the native particle, thus suggesting a different capsid organization. To summarize, GII.4 VLPs may be suitable for mimicking the behavior of HuNoVs with the exception of particle size and stability of BP below 72 °C. Thus, VLPs can be considered as relevant surrogates of HuNoVs when inactivation treatments induce significant changes in the capsid integrity, as described for some disinfectants^20,51^ and high-pressure processing^29^.

Bearing in mind the aforementioned limitations of VLPs, it is possible to discuss the relevance of HBGA-binding assays to reduce the over-estimation of the number of potential infectious HuNoVs given by the genome detection method. Substantial advantages of HBGA-binding approach can be suggested. Interactions between HuNoVs and HBGAs are very specific for numerous genotypes of HuNoVs. Although their role is not clearly defined in the host-cell infection process, HuNoV-HBGA interactions could be correlated with infectivity in inactivation assays^18^. HBGA-binding to the BP provides evidence of the presence of HuNoV capsids as it was confirmed by the lack of action of RNase A on the viral genome, suggesting persistence of potentially intact capsids. Conversely, this approach has several limitations that should be highlighted. The first point concerns methodological aspects. The specificity of HuNoV-HBGA interactions is dependent not only on the HBGA type (as previously demonstrated^39^), but also on the ionic strength of the media even though it does not disturb these interactions. The impact of the ionic strength is critical when comparing two different environmental media. Different interactions may be explained by the global charge of the viral particle. Considering the isoelectric point of GII.4 VLPs estimated to be between pH 5 and 6^44^, the global charge of the particles at pH 7.4 is negative under the tested conditions. By increasing the ionic strength, the electric double layer of viral particles could be compressed leading to a decrease in repulsive electrostatic interactions and an increase in hydrophobic interactions between VLPs and HBGAs. Dika *et al*.^52^ pointed out this complex interplay by showing the adhesion features of different viral surrogates (i.e. FRNAPH) to abiotic surfaces as a function of ionic strength.

The second point is related to a more rapid reduction in the number of HuNoVs able to bind to human HBGAs (T_90_ estimated at 166 days) compared to the whole encapsidated HuNoVs at 20 °C (T_90_ estimated at 294 days). This result indicates that at least two kinds of HuNoV particles are present, both having a capsid (as shown by the resistance to RNase A) and a genome but only one being capable of binding to HBGAs. Even though there is no evidence that the latter are really infectious, the use of HBGA-binding assays may reduce the over-estimation resulting from solely using the genome detection method. Our results are consistent with a previous study describing the persistence of GII HuNoV genomes in drinking water at 21 °C since the T_90_ was estimated at 298 days using a non-linear model^10^. Seitz *et al*.^53^ have also shown that the NV remained infectious in groundwater at room temperature in the dark for 61 days and the capsid remained intact for at least 427 days. Finally, HuNoV capsids may specifically bind to HBGAs when inactivated particles have a structural integrity of the capsid but an altered genome, or an intact genome with capsid damage(s) located in surrounding regions of the BP.

For a full evaluation of the HBGA-binding strategy, experimental data are required to correlate the loss of HuNoV binding to human HBGAs with the loss of infectivity after inactivation treatments when cellular models for the *in vitro* replication of HuNoVs will be available for routine application. The use of human HBGAs to evaluate the capsid integrity of HuNoVs according to the inactivation treatments needs more comprehensive studies for improving our understanding focused on HuNoV-HBGA interactions and the structural changes in the capsid proteins. Further researches are underway to explore the molecular modifications occurred on the BP but also on the overall capsid during these inactivation treatments.

## Materials and Methods

### Preparation of GII.4 VLP and GII.4 HuNoV samples

GII.4 VLPs (Cairo4 strain, a 2007-Osaka variant) were kindly provided by the National Reference Center for Gastroenteritis Viruses (NRCgev, Dijon, France). Production and purification of GII.4 VLPs were performed using the baculovirus-expressed VP1 system, as described previously^37,54^. For all experiments, purified GII.4 VLPs were prepared at a final concentration of 100 µg/mL in TNC buffer using the NanoDrop 2000 spectrophotometer (Thermo Fisher Scientific, Waltham, MA, USA). The working suspensions were kept at 4 °C before use.

GII.4 HuNoVs from human stools were genotyped^55^ and kindly provided by the NRCgev (Dijon, France). The purification of GII.4 HuNoVs was performed using a method described previously^56^, with significant modifications. Briefly, 1 g of fecal suspensions of GII.4 HuNoVs was added to 10 mL of 150 mM PBS solution at pH 7.4. Samples were clarified by addition of 1/3 (v/v) of chloroform, vortexed for 60 sec and centrifuged at 2,500 × *g* for 5 min. Supernatant was submitted to a second clarification step as described previously. The clarified samples were purified by dialysis using Float-A-Lyzer G2 with Biotech Cellulose Ester membranes (MWCO: 100 kD, volume: 1 mL, Spectra/Por®, Spectrum Laboratories, CA, USA). The membranes were dialyzed in a tank containing 10 L of 10 mM PBS at 4 °C overnight with gentle stirring. Dialysate samples were recovered and filtered using a 0.22 µm cellulose acetate membrane. After each step, the loss of HuNoV genomes was controlled. The viral genomes from 100 µL of purified HuNoV suspensions were extracted directly using the NucliSENS^®^ easyMAG^®^ kit (bioMérieux, Marcy-l’Etoile, France). The HuNoV genomes were detected using an RNA UltraSense One-Step quantitative RT-PCR system (Life Technologies, Carlsbad, CA, USA), according to the ISO 15216-1 standard recommendations^13^. Cq values were determined using CFX Maestro software (BioRad, Hercules, CA, USA). Quantification of HuNoV gc was performed using a standard curve of plasmids with a concentration range of 5 to 10^5^ gc/reaction mixture. Until use, the purified HuNoV suspensions were stored at 4 °C in the dark at a final concentration of 2.10^6^ gc/mL.

### Preparation and determination of HBGA types in human saliva

Saliva samples from 27 healthy adult volunteers were collected in 50 mL sterile polypropylene tubes. The saliva samples used in this study were described previously following approval by the Nantes University Hospital Review Board (study number BRD02/2-P)^37^, and informed consent was obtained from all donors. All experiments using saliva were performed in accordance with relevant guidelines and regulations. After harvesting, saliva samples were boiled at 100 °C for 5 min and then centrifuged at 10,000 × *g* for 5 min. Supernatants were stored at −20 °C until use. The presence of A, B, O blood group antigens and Lewis antigens in saliva samples at a dilution of 1:1,000 in 150 mM PBS was determined by ELISA, as described previously^22^.

### Natural ageing at 20 °C of GII.4 VLPs and HuNoVs

One mL of 10 µg/mL purified GII.4 VLPs in 10 mM and 150 mM PBS solutions and 1 mL of 2.10^6^ gc/mL of purified GII.4 HuNoVs in 10 mM PBS solution were exposed to natural ageing at 20 °C. This treatment, corresponding to the application of circadian cycles with a 24 h rotation of natural light and obscurity between October 2018 and July 2019, was evaluated over 105 days and 294 days for VLPs and HuNoVs, respectively. To that end, 20 separate 1 mL aliquots of VLPs and HuNoVs were placed in 2 mL Protein LoBind tubes. These aliquots were derived from a unique stock solution of 20 mL of VLPs and HuNoVs. For each sample, four and two analyses were performed for VLPs and HuNoVs, respectively.

### Heat treatments of GII.4 VLPs and HuNoVs

One mL of 10 µg/mL purified GII.4 VLPs and 1 mL of 2.10^5^ gc/mL of purified GII.4 HuNoVs in 150 mM PBS solution were separately exposed at 50 °C, 60 °C, and 72 °C for 30 min. Before adding 100 µL of 100 µg/mL GII.4 VLPs or 2.10^6^ gc/mL of GII.4 HuNoVs in borosilicate 4.9 glass tubes (thickness: 0.4 mm, diameter: 12 mm, Fiolax, Schott AG, Mitterteich, Germany), 900 µL of 150 mM PBS solution was pre-warmed in a glycol bath GR150 S5 (Grant Instruments, Cambridge, UK) to minimize the effect of the temperature rise. After heating, treated samples were immediately placed on ice to stop the reaction. The temperature was monitored in the glycol bath but also in a control tube containing 1 mL of 150 mM PBS solution using temperature probes (SSA-TF, E-Val™ Pro, ELLAB, Hillerød, Denmark) coupled to a data logger (Valsuite™ Basic software, ELLAB, Hillerød, Denmark). Experiments were performed in triplicate for VLPs and HuNoVs under the same conditions.

### GII.4 VLP binding to saliva samples: HBGA-binding ELISA

HBGA-binding ELISA was performed using the method described previously^22^, with minor adjustments. Briefly, 100 µL of supernatant of saliva samples of known HBGA types were coated in 96-well microtiter plates at a dilution of 1:1,000 in 150 mM PBS at 4 °C overnight. Negative controls were systematically added using saliva-coated wells treated with sodium periodate as described previously^42^, with slight modifications. After blocking with 200 µL of 5% non-fat dried milk at 37 °C for 2 h, 100 µL of native and treated GII.4 VLPs diluted in PBS solutions was incubated at 37 °C for 1 h. The VLP binding to the saliva samples was detected with anti-GII.4 VLP MAbs kindly provided by the NRCgev (Dijon, France) at a 1:10,000 dilution in 150 mM PBS for 1 h at 37 °C, followed by the addition of horseradish peroxidase (HRP)-conjugated goat anti-mouse IgG (Chemicon, Merck Millipore, Burlington, MA, USA) at a dilution of 1:30,000 in 150 mM PBS for 1 h at 37 °C. Between each step, the wells were washed three times with 200 µL of 150 mM PBS containing 0.05% Tween 20 using a microplate washer (Elx50, BioTeK Instruments, Winooski, VT, USA). The enzyme activity was detected using the TMB kit (Pierce Biotechnology, Rockford, IL, USA). After incubation for 15 min at room temperature, the reaction was stopped by adding 100 µL of 1 M sulfuric acid. The OD values of the enzyme activity were read at 450 nm with an enzyme immunoassay spectrum reader (Elx800, BioTeK Instruments, Winooski, VT, USA). All samples were analyzed in duplicate. Inconsistent results were rejected if the gap between OD_450_ value duplicates was above 0.1. To give confidence, the P/N ratio was calculated by dividing the mean OD_450_ values of the sample by the mean OD_450_ values of the negative control. When P/N ratio was above 2, results were considered positive. When P/N ratio was below 2, results were considered negative^57^.

The sensitivity and the linearity of the HBGA-binding ELISA method were determined using the supernatant of OLe^+^ saliva sample and various contents of GII.4 VLPs (ranging from 0.05 and 10.0 µg/mL) diluted in 10 mM and 150 mM PBS solutions. The experiments were performed in four replicates.

### GII.4 HuNoV binding to saliva samples: HBGA-binding assays followed by RNA amplification by RT-qPCR

This method was performed using a combination of approaches described previously^22,56^. Briefly, supernatant of OLe^+^ saliva sample was coated in 96-well microtiter plates as described above. Negative controls were also added using the same saliva sample treated with sodium periodate. After blocking with non-fat dried milk, 100 µL of native and treated GII.4 HuNoV suspensions, previously treated by RNase A^58^, was incubated at 37 °C for 1 h. Between each step, the wells were washed three times as described above. The bound capsid proteins of HuNoVs were lysed directly in the wells using 150 µL of a buffer containing 5 M of guanidium thiocyanate for 10 min at room temperature. For each condition (native or treated HuNoVs, negative controls), 10 wells were pooled on the same 96-well microtiter plate to analyze 1 mL of purified HuNoVs. The viral genomes were purified and detected as described previously. The HuNoV genomes were also detected after RNase A treatment. The LOD was determined at 200 gc/mL and the LOQ was determined at 1,000 gc/mL.

### TEM observations

GII.4 VLPs exposed to heating were observed by TEM after negative staining with phosphotungstic acid. Briefly, 10 µL of heated GII.4 VLPs were absorbed to carbon coated copper grids by incubating at room temperature for 3 min. The excess was eliminated and the copper grids were negatively stained with 2% phosphotungstic acid by incubating at room temperature for 1 min. The stained copper grids were air dried for 3 min and then observed by TEM.

### Statistical analysis

All statistical analyses were performed using R statistical software (Rx64 v.3.5.3). The Shapiro-Wilk test was performed to check normality of the data with alpha level of 0.05. If data set followed a normal distribution (*p* > 0.05), parametric tests were applied. A paired or unpaired sample Student’s *t*-test was performed for dependent or independent data following a normal distribution, respectively. Non-parametric tests were used for data sets having a non-normal distribution (*p* < 0.05). For dependent or independent data with non-normal distribution, a Wilcoxon signed-rank test or a Mann-Whitney U test was applied. For all tests, the significance level was set to 0.05.

## Supplementary information

supplementary info

## Data Availability

All data analyzed in this study are included in this published article and also its Supplementary Information files.
