# Supplementary material for: Effect of natural ageing and heat treatments on GII.4 norovirus binding to Histo-Blood Group Antigens
Source: Sci Rep. 2019 Oct 25;9:15312. doi: 10.1038/s41598-019-51750-4 (PMC6814753; doi:10.1038/s41598-019-51750-4)
Supplement: Supplementary file 1 — supplementary info [file 41598_2019_51750_MOESM1_ESM.docx]

**Effect of natural ageing and heat treatments on GII.4 norovirus binding to Histo-Blood Group Antigens**

**Maëlle Robin^1^, Manon Chassaing^1,2^, Julie Loutreul^1^, Alexis de Rougemont^3,4^, Gaël Belliot^3,4^, Didier Majou^5^, Christophe Gantzer^2^ & Nicolas Boudaud^1*^**

^1^Actalia, Food Safety Department, F-50000 Saint-Lô, France.

^2^LCPME, UMR 7564 CNRS, University of Lorraine, F-54601 Villers-lès-Nancy, France.

^3^National Reference Centre for Gastroenteritis Viruses, Laboratory of Virology, University Hospital of Dijon, F-21000 France.

^4^UMR PAM A 02.102 Food and Microbiological Processes, University of Bourgogne Franche-Comté / AgroSup Dijon, F-21000 France.

^5^ACTIA, F-75231 Paris Cedex 05, France.

^*^Corresponding author: [n.boudaud@actalia.eu](mailto:n.boudaud@actalia.eu)

**
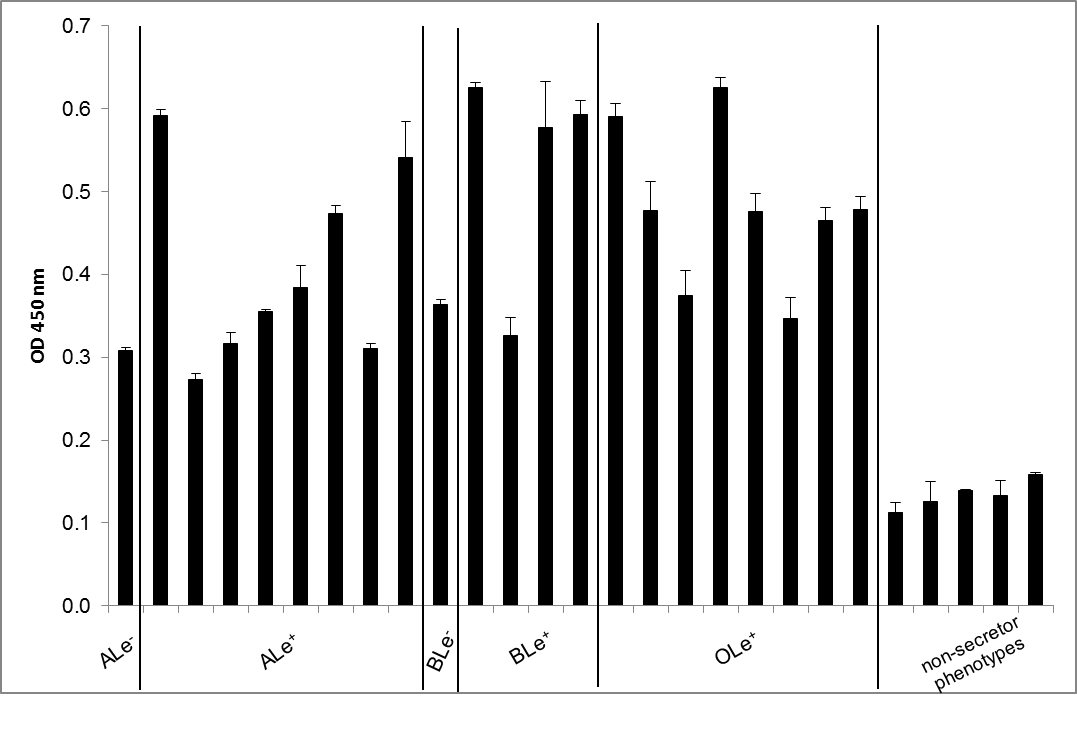
**

**Supplementary Figure S1. Binding profile of GII.4 VLPs to all human saliva samples.** The OD_450_ values were obtained at 1 µg/mL of GII.4 VLPs in 150 mM PBS solution by HBGA-binding ELISA. Each data point represents the OD_450_ mean of two replicates and error bars indicate standard deviations.

**
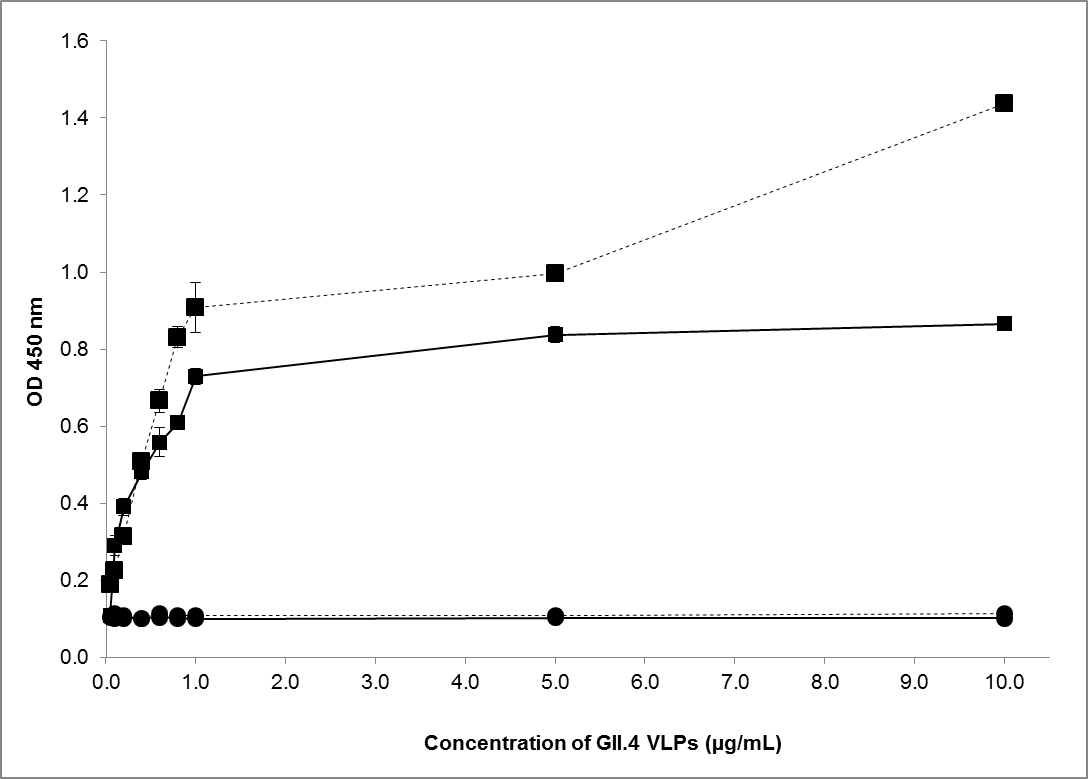
**

**Supplementary Figure S2. Sensitivity and linearity of the HBGA-binding ELISA assays.** The sensitivity and the linearity of the HBGA-binding ELISA method were determined using OLe^+^ human saliva and various concentrations of GII.4 VLPs ranging from 0.05 and 10.0 µg/mL in 10 mM PBS solution (solid line) and 150 mM PBS solution (dashed line). Squares (■) and circles (●) represent the VLP-binding to untreated and sodium periodate-treated OLe^+^ human saliva, respectively. Each data point represents the OD_450_ mean of four replicates and error bars indicate standard deviations.
